# Exo70 is an independent prognostic factor in colon cancer

**DOI:** 10.1038/s41598-017-05308-x

**Published:** 2017-07-11

**Authors:** Li Xiao, Kaifeng Zheng, Xia Lv, Jihuan Hou, Liang Xu, Yujie Zhao, Fei Song, Yaqiong Fan, Hanwei Cao, Wenqing Zhang, Xiaoting Hong, Yan-yan Zhan, Tianhui Hu

**Affiliations:** 1 Cancer Research Center, Xiamen University Medical College, Xiamen, 361102 Fujian Province P.R. China; 2 Department of Oncology, Zhongshan Hospital Affiliated to Xiamen University, Xiamen, 361004 Fujian Province P.R. China

## Abstract

Exo70, a key component of the Exocyst complex, plays important roles in human cancer progression beyond exocytosis. However, the expression of Exo70 and its prognostic value for patients with colon cancer has not been well investigated to date. In this study, we observed that the mRNA and protein levels of Exo70 were upregulated in 11 of 13 colon cancer tissues, compared with their normal counterparts, which was validated by immunohistochemical analysis in a tissue microarray containing 89 pairs of colon cancer tissues and the matched adjacent normal tissues. Statistical analysis revealed that Exo70 expression is positively correlated with tumor size, invasion depth, TNM stage and distant metastasis. Kaplan-Meier survival analysis showed that colon cancer patients with higher Exo70 expression have a poorer clinical outcome than those with lower Exo70 expression. Multivariate Cox regression analysis revealed that Exo70, age and distant metastasis were there independent prognostic factors for overall survival rate of colon cancer patients. Through gain- and loss of Exo70 in colon cancer cells, we found that Exo70 could enhance the migration ability of colon cancer cells. Taken together, our studies revealed that Exo70 might be a promising negative prognostic factor and a potential therapeutic target for colon cancer.

## Introduction

Colorectal cancer is the third most commonly diagnosed cancer globally, accounting for 10.0% of the estimated 14.1 million new cancer cases^1–3^. Moreover, it is the third leading cause of cancer-related death in women and the fourth in men, with 693,600 deaths occurring worldwide^4, 5^.

Colorectal cancers vary in terms of risk factors and clinical and biological characteristics based on their location within the colon or rectum, suggesting distinct etiologies and carcinogenic mechanisms^6, 7^. Despite a substantial rise in survival over the last two decades, the 5-year disease-specific overall survival rate is approximately 59% for colon cancer^8^. The prognosis of colon cancer patients is still extremely poor because of distant metastasis and recurrence. Therefore, novel therapeutic approaches and prognostic factors are required to improve the poor prognosis of colon cancer patients.

Exo70 is a critical component of the evolutionarily conserved Exocyst complex, a heterooctamer composed of Sec3, Sec5, Sec6, Sec8, Sec10, Sec15, Exo70 and Exo84^9^. Exocyst is essential for exocytosis via tethering secretory vesicles to specific domain of the plasma membrane^10–12^. Recent studies suggested that Exo70 and the Exocyst complex are implicated in several key stages of cancer and may provide a link between tumorigenesis and cancer dissemination in melanoma and breast cancer^13–17^. Exo70 plays important roles in cell invasion by mediating the secretion of MMPs at focal degrading sites and regulating Arp2/3-mediated actin dynamics^15–17^. Isoform switching of Exo70 plays an important role in the transition between the epithelial and mesenchymal states^17^. However, the diagnostic and prognostic significance of Exo70 expression and its association with clinicopathologic features have not yet been reported in colon cancer.

In this study, we had conducted a comprehensive evaluation of Exo70 expression by real-time polymerase chain reaction (PCR) analyses, western blot and immunohistochemistry in colon cancer and to systematically elucidate the prognostic relevance of Exo70 expression and clinicopathologic features. Gain- and loss-of Exo70 in colon cancer cell lines suggested that Exo70 promoted the cellular migration. We provided evidence that Exo70 was associated with the invasion and metastasis of colon cancer and our results indicated that Exo70 might serve as a prognostic factor in colon cancer.

## Results

### Differential expression of Exo70 in colon cancer tissues and the matched adjacent normal colon tissues

**Figure 1:**
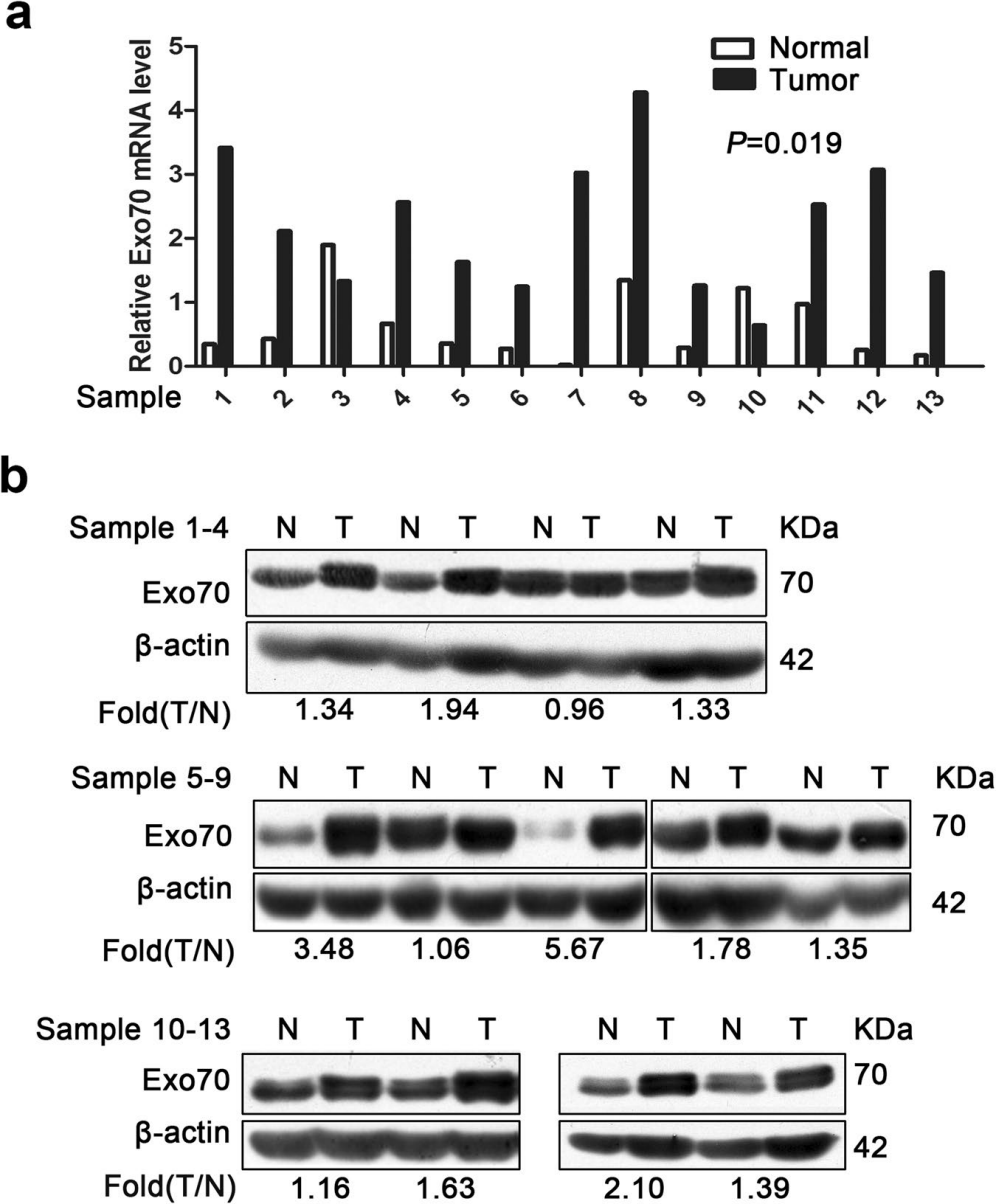
Real-time PCR and western blot analyses of Exo70 expression in 13 pairs of resection specimens from colon cancer patients. (**a**) Relative Exo70 mRNA levels normalized with GAPDH. Relative expression levels of Exo70 mRNA are upregulated in colon cancer tissues compared with noncancerous colon tissues (*P* < 0.05). The relative expression data were analyzed by the 2^−ΔΔCt^ method. GAPDH was used as an internal control. (b) Results of western blot analysis of Exo70 protein expression in 13 pairs of resection specimens from colon cancer patients. The β-actin was used as loading control.

To investigate the expression of Exo70 at mRNA level, we detected the mRNA expression of Exo70 in 13 pairs of fresh colon cancer tissues and the matched adjacent normal tissues. Our real-time PCR analysis indicated that in colon cancer patients, Exo70 mRNA was significantly increased in 84.6% (11/13) tumor tissues compared with the matched non-tumor tissues (2.20 ± 1.05 *versus* 0.63 ± 0.56; *P* = 0.019; Fig. 1a). We then determined the expression of Exo70 at protein level in these 13 pairs of colon cancer tissues and the adjacent normal tissues. We also evaluated the densitometry of western blot, and the ratio of tumor/normal (Exo70 protein expression) showed that Exo70 protein was greatly increased in 84.6% (11/13) of colon cancer tissues (Fig. 1b).

**Figure 2:**
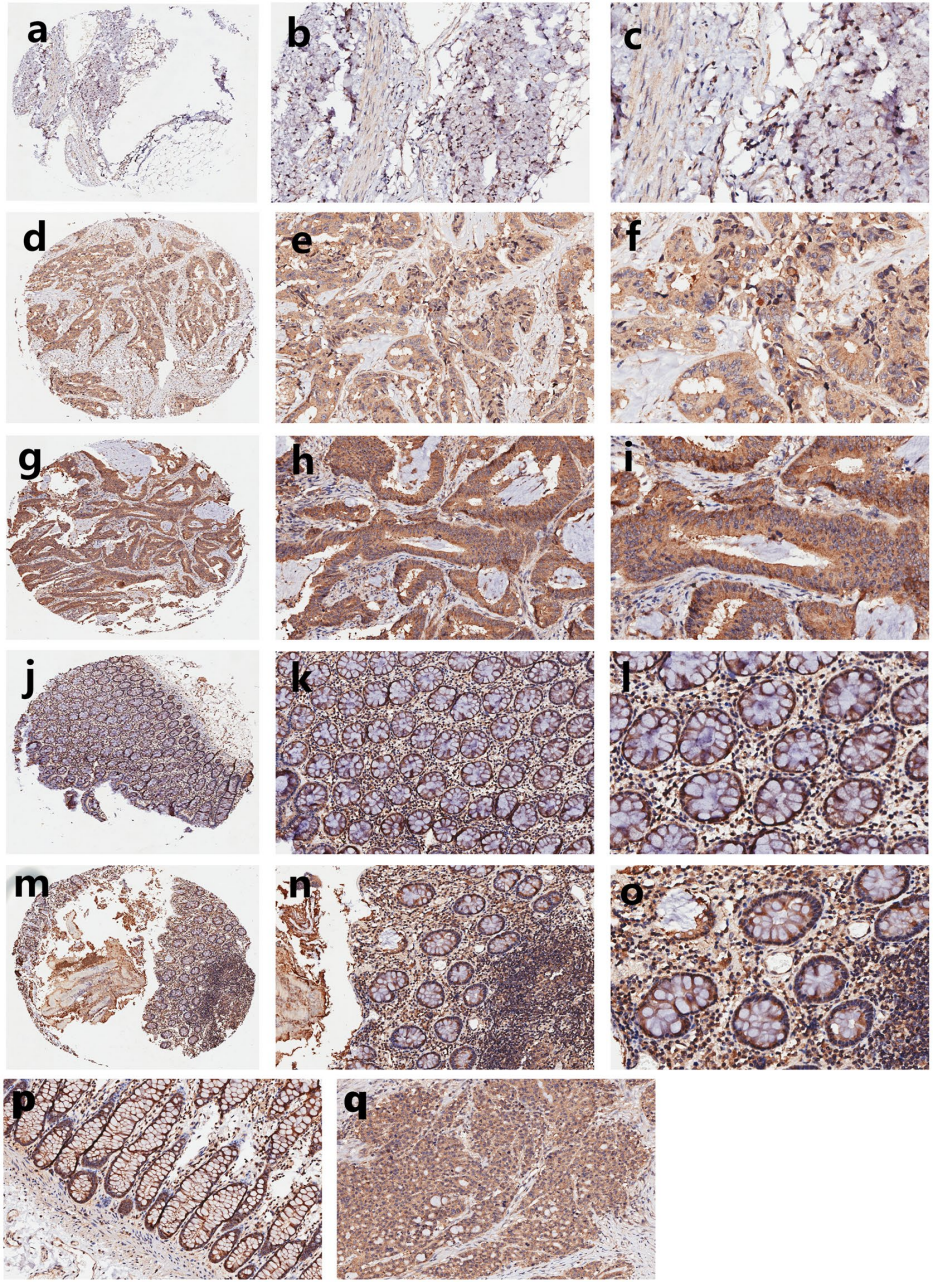
Representative images of Exo70 expression in colon cancer tissues and corresponding noncancerous tissues. (**a**–**c**) Slightly positive (+) of Exo70 expression in colon cancer tissue samples. (**d**–**f**) Moderately positive (++) of Exo70 expression in colon cancer tissues. (**g**–**i**) Strongly positive (+++) of Exo70 expression in colon cancer tissues. (**j**–**l**) Slightly positive (+) of Exo70 expression in noncancerous tissues. (**m**–**o**) Moderately positive (++) of Exo70 expression in noncancerous tissues. Original magnification x40 (**a**,**d**,**g**,**j** and **m**), x200 (**b**,**e**,**h**,**k** and **n**), and x400 (**c**,**f**,**i**,**l** and **o**). (**p**–**q**) Representative images of Exo70 expression in one patient side by side with paired noncancerous tissue and colon cancer tissue. Noncancerous tissue is slightly positive (+) (**p**, original magnification x200), and colon cancer tissue is strongly positive (+++) (**q**, original magnification x200).

**Figure 3:**
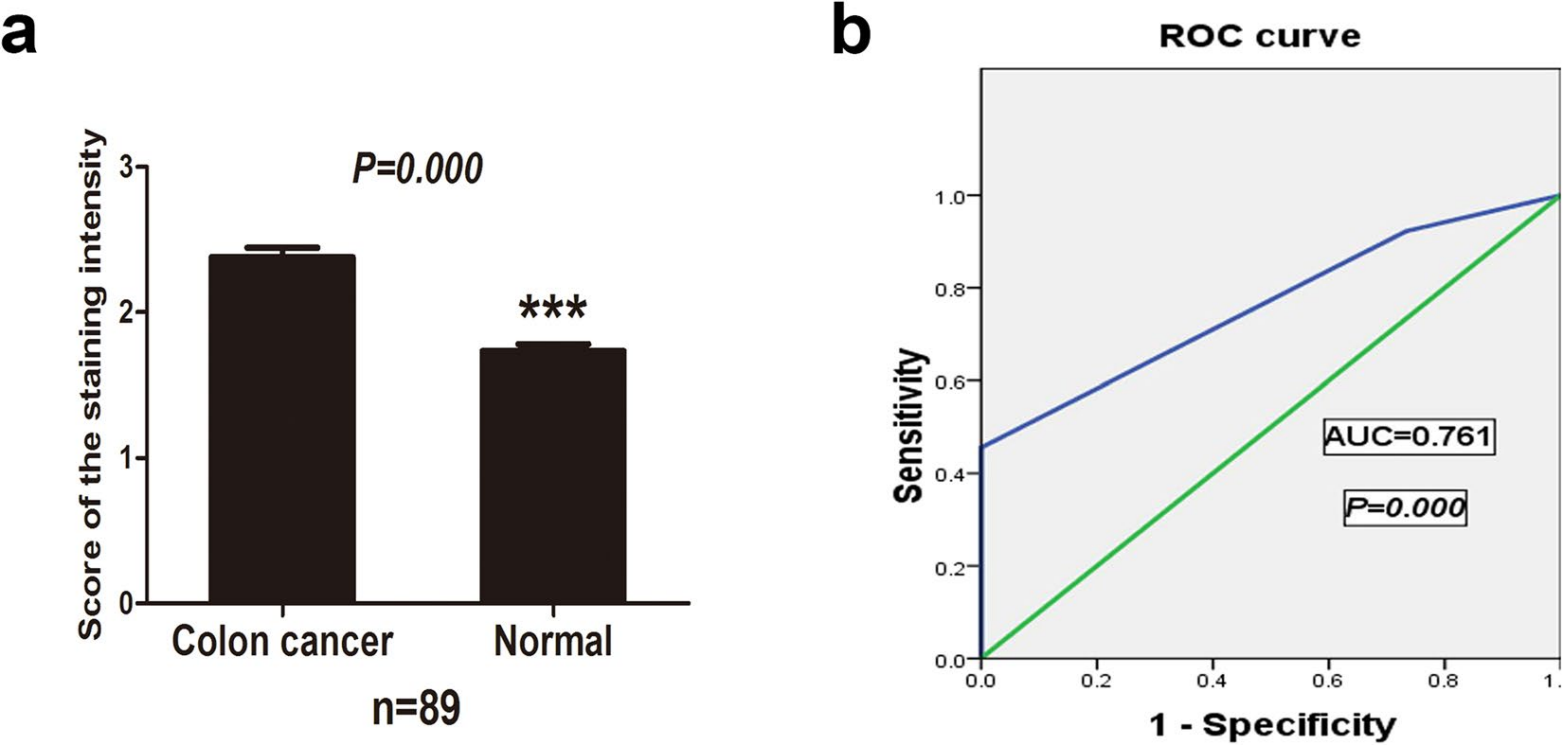
Expression levels of Exo70 are upregulated in colon cancer tissues compared with noncancerous colon tissues and ROC curve analysis using Exo70 for discriminating the colon cancer from noncancerous colon tissues. (**a**) Significant differences in Exo70 expression between the colon cancer and adjacent normal colon tissues (***P < 0.0001). (**b**) ROC curve were plotted by sensitivity and specificity for Exo70 immunohistochemical staining (*P* < 0.0001). ROC, receiver operating characteristic.

The expression of Exo70 in colon cancer was further evaluated in the specimens from additional 89 patients by immunohistochemical analysis. The specificity of the commercial anti-EXO70 antibody was firstly verified by western blot and immunohistochemistry (IHC) in EXO70 knocking-down (KD) colon cancer cells (Supplementary Fig. S1a,b). Exo70 expression was detectable in all these tumor tissues and tumor-adjacent nontumor tissues. We first confirmed the location of Exo70 in these tissues. As shown in Fig. 2, Exo70 was predominantly located in the cytoplasm in both normal colonic epithelial cells and colon cancer cells. Strongly positive (+++) Exo70 expression was detected in 40 colon cancer tissues (44.9%) but in none of the 89 adjacent nontumor tissues (0.0%), moderately positive (++) Exo70 expression was detected in 42 colon cancer tissues (47.2%) and in 65 adjacent nontumor tissues (73.0%), slightly positive (+) Exo70 expression was detected in 24 adjacent nontumor tissues (27.0%) and in only 7 colon cancer tissues (7.87%) (Table 1). Therefore, IHC revealed that Exo70 expression was higher in colon cancer tissues than in the adjacent normal tissues (*P* = 0. 000, Fig. 3a).

**Table 1:** Expressions of Exo70 in the adjacent normal mucosa and colon cancer tissues by IHC.

Group	Exo70 expression (immuneohistochemical staining)			*P*
	**+**	**++**	**+++**	
Adjacent nontumor Tissues	24(27.0%)	65(73.0%)	0(0.0%)	<0.001
Colon cancer tissues	7(7.9%)	42(47.2%)	40(46.1%)

Based on the Exo70 IHC staining scores for each sample and the cut‑off score determined by ROC curve analysis, evaluation score of the reactive cells was calculated by the intensity score and its proportion score. ROC curve analysis was performed to assess the sensitivity and specificity of Exo70 expression for distinguishing the colon cancer from nontumor tissues, which was presented as the value of area under the curve (AUC) in Fig. 3b. For distinguishing colon cancer tissues from nontumor tissues, the AUC of Exo70 was 0.761 (95% CI = 0.692–0.831, *P* = 0.000). The ROC curves for Exo70 clearly show the point on the curve closest to (0.0, 1.0), which maximizes both sensitivity and specificity. The sensitivity was 92.2% and the specificity was 73.3%. The Exo70 IHC cut-off scores for distinguishing the colon cancer from nontumor tissues were 5.0. Our findings suggested that Exo70 had higher sensitivity and specificity in distinguishing the colon cancer from nontumor tissues.

### Correlation between Exo70 expression and clinicopathologic parameters of patients with colon cancer

The association between Exo70 expression and the clinicopathologic parameters is shown in Table 2. Higher expression of Exo70 was found to be significantly associated with distant metastasis (*P* = 0.015) and advanced TNM stage of disease (*P* = 0.025). In addition, higher expression of Exo70 was found to be associated with enhanced tumor size (*P* = 0.042) and tumor invasion depth (*P* = 0.04). No significant association was observed between Exo70 expression and patient’s gender, patient’s age, tumor position, tumor differentiation, lymphovascular invasion and lymph node metastasis.

**Table 2:** Correlation between Exo70 expression and clinicopathological features of colon cancer patient (n = 89).

Characteristic	N	Exo70 immunohistochemical staining			*P*
		**+**	**++**	**+++**	
Gender					0.666
Male	46	3(6.5%)	23(50.0%)	20(43.5%)	
Female	43	3(7.0%)	19(44.2%)	21(48.8%)	
Age(years)					0.786
≤68	44	28(63.6%)	11(25.0%)	5(11.4%)	
>68	45	5(11.1%)	34(75.6%)	6(13.3%)	
Position					0.147
Left colon	40	5(12.5%)	14(35.0%)	21(52.5%)	
Right colon	36	4(11.1%)	20(55.6%)	12(33.3%)	
Tumor size					0.042^a^
≤3 cm	10	2(20.0%)	6(60.0%)	2(20.0%)	
>3 cm	79	4(5.1%)	36(45.6%)	39(49.4%)	
Tumor differentiation					0.641
Well	7	2(28.6%)	2(28.6%)	3(42.9%)	
Moderately	48	2(4.2%)	25(52.1%)	23(47.9%)	
Poorly	34	4(11.8%)	15(44.1%)	15(44.1%)	
Ivasion depth					0.040^a^
T1-T2	10	4(40.0%)	3(30.0%)	3(30.0%)	
T3-T4	79	5(6.3%)	39(49.4%)	35(44.3%)	
Lymphovascular invasion					0.497
No	71	20(27.4%)	27(38.0%)	24(33.8%)	
Yes	17	5(29.4%)	6(35.3%)	6(35.3%)	
Lymph node metastasis					0.719
N(−)	55	5(9.1%)	24(43.6%)	26(47.3%)	
N(+)	34	2(5.9%)	18(5.3%)	14(41.2%)	
Distant metastasis					0.015^a^
M0	78	6(7.7%)	42(53.8%)	30(38.5%)	
M1	11	0(0.0%)	2(18.2%)	9(81.8%)	
TNM stage(AJCC)					0.025^a^
Stage I	8	1(12.5%)	3(37.5%)	4(50.0%)	
Stage II	39	4(3.2%)	18(46.2%)	17(43.6%)	
Stage III	31	1(3.2%)	19(61.3%)	11(35.5%)	
Stage IV	11	0(0.0%)	2(18.2%)	9(81.8%)	

^a^Statistically significant.

### High Exo70 expression correlates with decreased survival in colon cancer patients

**Figure 4:**
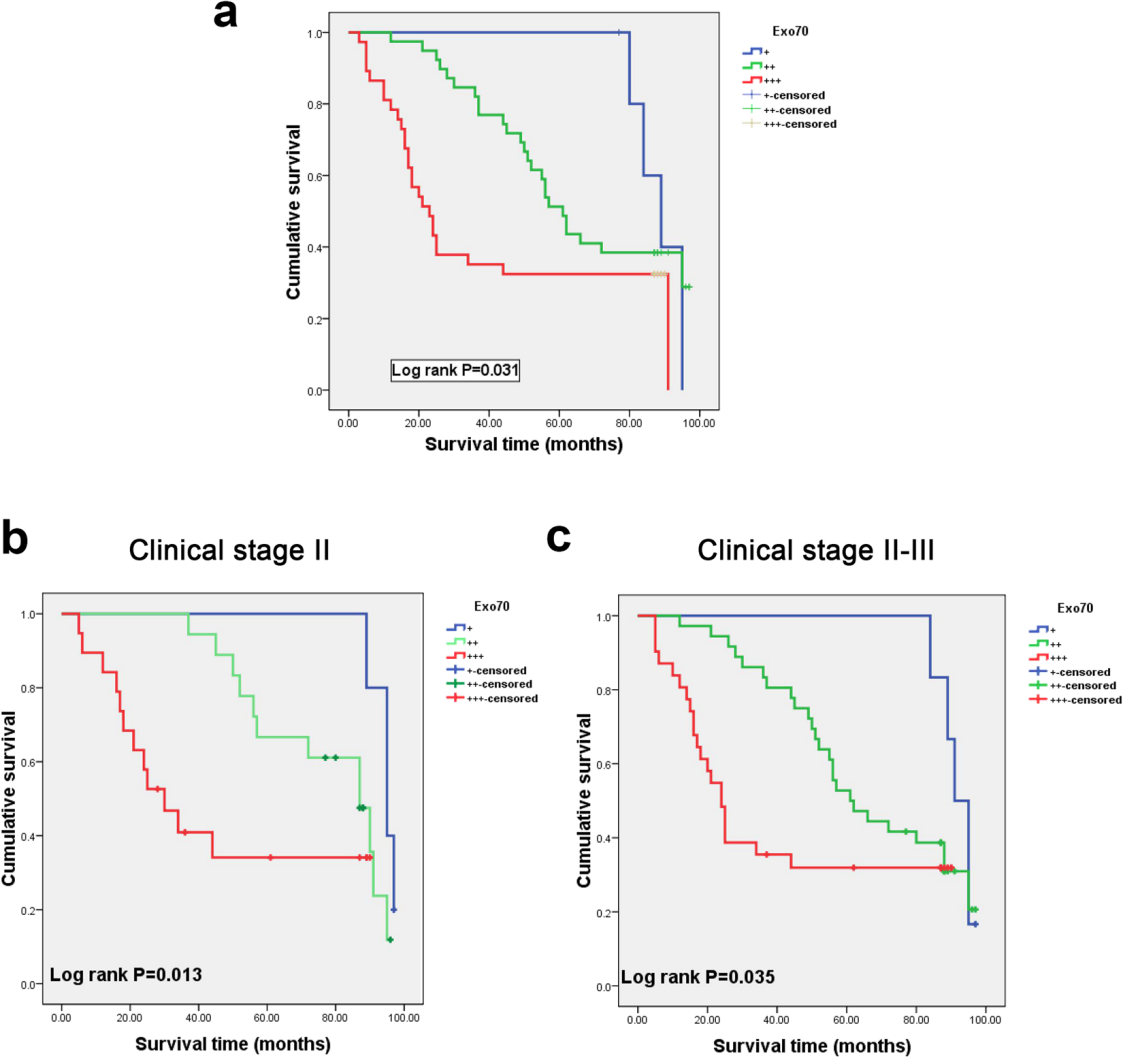
Exo70 expression directly correlates with patient’s survival. (**a**) Kaplan Meier graph depicting relation between overall survival and Exo70 staining incolon cancer patients (n = 89). (**b**,**c**) Subgroup survival analysis of stage II alone and stage II + III. Slightly positive (+), moderately positive (++), strongly positive (+++), *P < 0.05.

To further define the role of Exo70 in colon cancer prognosis, a Kaplan-Meier analysis of OS showed reduced survival in patients with strongly positive Exo70 expression (+++) compared to patients with slightly positive (+) and moderately positive (++) Exo70 expression (log-rank, *P* = 0.03, Fig. 4a). The results showed that Exo70 expression was dramatically associated with colon cancer patients’ overall survival; the mean OS was 88.6 months in the slightly positive (+) Exo70 expression group and 65.1 months in the moderately positive (++) Exo70 expression group, whereas it was only 41.1 months in the strongly positive (+++) Exo70 expression group. Besides, we also assessed the correlation between Exo70 expression and overall survival in different sub-groups (Clinical stage II and Clinical stage II-III) according to TNM stage. It turned out that the overall survival is poor in Colon cancer patients with increased Exo70 expression, regardless of TNM stage (Fig. 4b,c).

To directly identify the risk factors associated with patients’ overall survival, Exo70 expression level as well as 11 clinicopathologic factors were evaluated by conducting Cox univariate and multivariate analyses. As a result, the univariate analysis indicated that expression of Exo70 (weak and moderate *vs* strong), Age (≤68 y *vs* >68 y), TNM stage (I, II and III *vs* IV), lymphatic metastasis (yes *vs* no) and distant metastasis (yes *vs* no) were significant prognostic factors for overall survival prediction (Table 3). Moreover, Table 4 presents the results of a multivariate Cox regression analysis of survival including age, distant metastasis, lymph node metastasis, and TNM stage. Multivariate analysis showed that Exo70 expression was the most significant independent risk factor (hazard ratios: 2.258, 95% CI:1.354–3.788, *P* = 0.002), followed by distant metastasis (hazard ratio: 2.440, 95% CI:1.189–4.902, *P* = 0.0015), older age (hazard ratios: 2.283, 95% CI:1.342–3.768, *P* = 0.003). This analysis confirmed that Exo70 expression, age, distant metastasis were independent predictors of colon patients’ overall survival. There were no significant association between lymph node metastasis and TNM stage with overall survival: lymph node metastasis (hazard ratio: 1.727, 95% CI:0.872–3.420, *P* = 0.117), TNM stage (hazard ratio: 0.808, 95% CI:0.414–1, 580, *P* = 0.534). These data indicated that high Exo70 expression may be a predictor for diagnosis and prognosis in colon cancer patients.

**Table 3:** Univariate analysis of the influence of various parameters on overall survival in colon cancer patients.

Variables	OS		
	Hazard ratio	95% CI	*P*
Age(y), ≤68 *vs* > 68	1.822	1.095–2.690	0.018^a^
Sex, male *vs* female	0.927	0.536–1.535	0.716
Tumor size(cm) ≤3 *vs* > 3	2.719	0.836–8.733	0.093
Tumor location, left *vs* right	1.339	0.758–2.365	0.315
Tumor differentiaton, well and moderate *vs* poor	1.126	0.704–1.800	0.621
Depth of invasion, T1-T2 *vs* T3-T4	0.923	0.395–2.160	0.854
Lymph node metastasis, yes *vs* no	1.832	1.234–2.720	0.003^a^
Distant metastasis, yes *vs* no	2.059	1.138–3.727	0.017^a^
TNM stage, I, II and III *vs* IV	1.648	1.086–2.500	0.019^a^
Exo70 expression, weak and moderate *vs* strong	1.717	1.095–2.690	0.018^a^

Abbreviations: OS, overall survival; CI, confidence interval. ^a^Statistically significant.

**Table 4:** Multivariate analysis of the influence of various parameters on overall survival in colon cancer patients.

Variables	OS		
	Hazard ratio	95% CI	*P*
Age(y), ≤68 *vs* > 68	2.283	1.342–3.768	0.003^a^
Distant metastasis, yes *vs* no	2.440	1.189–4.902	0.015^a^
Lymph node metastasis, yes *vs* no	1.727	0.872–3.420	0.117
TNM stage, I, II and III *vs* IV	0.808	0.414–1.580	0.534
Exo70 expression, weak and moderate *vs* strong	2.258	1.354–3.768	0.002^a^

Abbreviations: OS, overall survival; CI, confidence interval. ^a^Statistically significant.

As the results above showed that Exo70 is a poor prognostic feature of colon cancer patients in clinical stage II and clinical stage II-III. To compare the prognostic power of Exo70 *vs* other poor prognostic features in Stage II and II + III, including T4 tumors (stage IIB/IIC); poorly differentiated histology; lymphovascular invasion *etc*. Cox univariate and multivariate analyses were conducted to evaluate Exo70 expression level as well as other poor prognostic features. Tables 5 and 6 presents the results of a univariate and multivariate Cox regression analysis of survival including age, sex, T stage, tumor differentiation, lymphovascular invasion and distant metastasis, lymph node metastasis, and Exo70 expression level in stage II and stage II + III colon cancer patients. As a result, the univariate analysis indicated that expression of Exo70 (weak and moderate *vs* strong), tumor differentiation (well and moderate *vs* poor), lymphovascular invasion (yes *vs* no) were significant prognostic factors for overall survival prediction in stage II colon cancer (Table 5). Multivariate analysis showed that tumor differentiation was the most significant independent risk factor (hazard ratios: 3.199, 95% CI:1.371–6.839, *P* = 0.016), followed by lymphovascular invasion (hazard ratios: 2.996, 95% CI:1.169–4.509, *P* = 0.023), Exo70 expression level (hazard ratios: 2.938, 95% CI:1.790–10.253, *P* = 0.025). The univariate analysis in Table 6 indicated that expression of Exo70 (weak and moderate *vs* strong), tumor differentiation (well and moderate *vs* poor), lymphovascular invasion (yes *vs* no) were also significant prognostic factors for overall survival prediction in stage II + III colon cancer. Multivariate analysis showed that lymphovascular invasion was the most significant independent risk factor (hazard ratios: 2.835, 95% CI:1.153–6.023, *P* = 0.022), followed by Exo70 expression level (hazard ratio: 1.685, 95% CI:1.057–2.685, *P* = 0.028). These data indicated that high Exo70 expression and lymphovascular invasion are predictors for poor prognosis in stage II and stage II + III colon cancer patients.

**Table 5:** Univariate and multivariate analysis of the influence of various parameters on overall survival in Stage II colon cancer patients. Abbreviations: OS, overall survival; CI, confidence interval. ^a^Statistically significant.

Variables	Univariate analysis			Multivariate analysis		
	HR	95% CI	*P*	HR	95% CI	*P*
Age(y), ≤68 *vs* > 68	1.780	0.645–4.908	0.265			
Sex, male *vs* female	1.229	0.444–3.452	0.692			
T stage, T3 *vs* T4	2.559	1.162–5.670	0.077			
Tumor Differentiaton, well and moderate *vs* poor	3.896	1.942–7.246	0.009^a^	3.199	1.371–6.839	0.016^a^
Exo70 expression, weak and moderate *vs* strong	3.635	1.428–9.154	0.013^a^	2.938	1.790–10.253	0.025^a^
Lymphovascular Invasion, yes *vs* no	3.646	1.607–8.275	0.012^a^	2.996	1.169–4.509	0.023^a^

**Table 6:** Univariate and multivariate analysis of the influence of various parameters on overall survival in Stage II + III colon cancer patients.

Variables	Univariate analysis			Multivariate analysis		
	HR	95% CI	*P*	HR	95% CI	*P*
Age(y), ≤68 *vs* > 68	1.981	0.973–5.128	0.156			
Sex, male *vs* female	1.887	0.854–4.326	0.176			
T stage, T2-T3 *vs* T4	1.213	0.564–2.986	0.685			
Tumor Differentiaton, well and moderate *vs* poor	3.061	1.244–6.887	0.022^a^	1.599	0.988–2.589	0.056
Exo70 expression, weak and moderate *vs* strong	2.241	1.145–6.987	0.035^a^	1.685	1.057–2.685	0.028^a^
Lymphovascular Invasion, yes *vs* no	2.498	1.853–7.894	0.032^a^	2.835	1.153–6.023	0.022^a^

Abbreviations: OS, overall survival; CI, confidence interval. ^a^Statistically significant.

### The effects of changed expression of Exo70 on the migration ability of colon cancer cells

**Figure 5:**
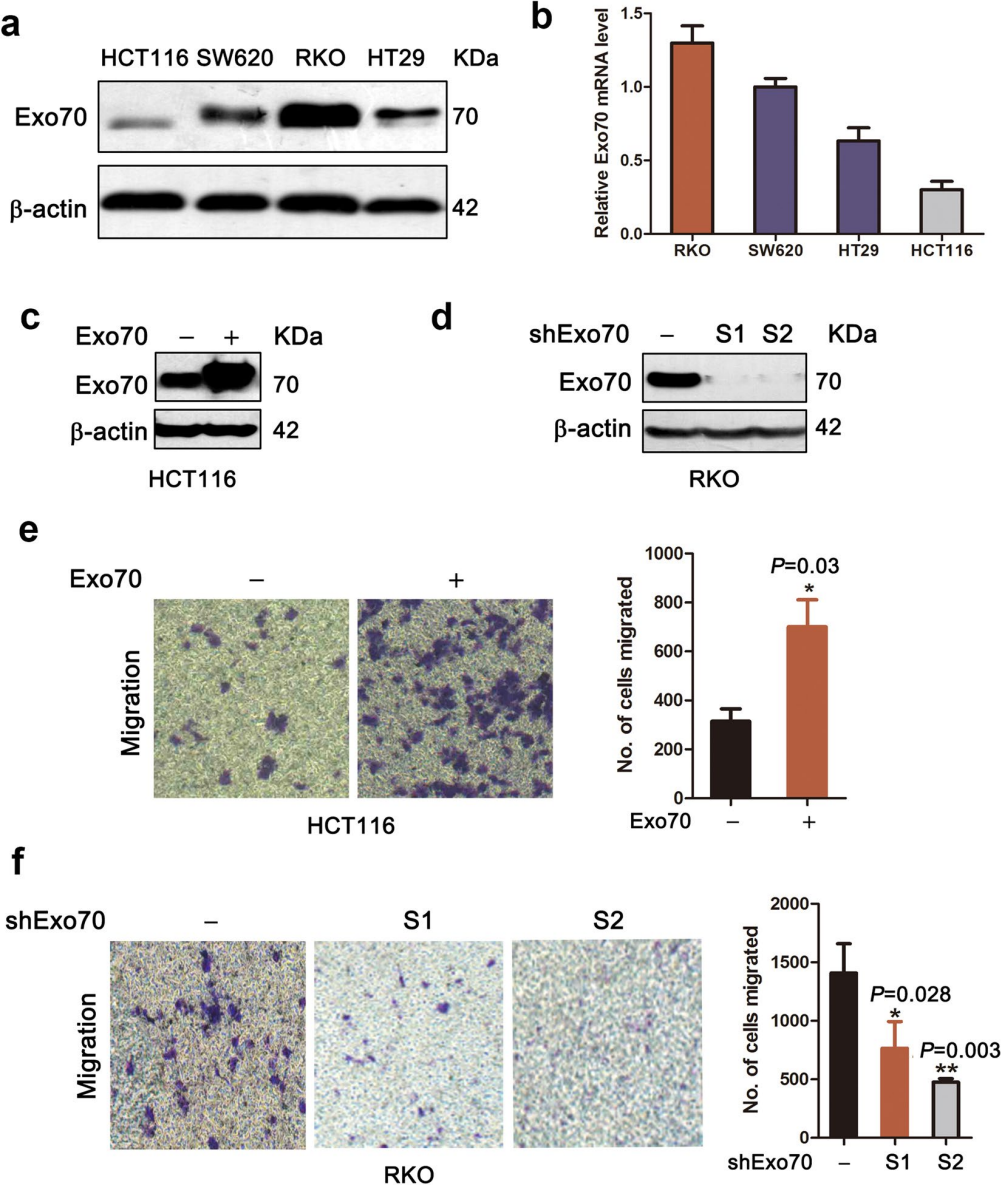
Exo70 enhanced migration of colorectal cancer cells. (**a**) Relative Exo70 protein levels in four colorectal cancer cell lines were determined by western blot analysis. (**b**) Relative mRNA levels of Exo70 in indicated cells were determined by real-time PCR. (**c**,**d**) Exo70 overexpression and knockdown were detected by western blot analysis. (**e**,**f**) Representative photographs of migratory cells on the membrane in transwell assays (200×). Average numbers of migrated cells. Data are represented as mean ± SD of three independent experiments. *P < 0.05; **P < 0.01.

To explore the role of Exo70 in colon cancer progression, four human colon cancer cell lines, including RKO, SW620, HT29 and HCT116, were employed to evaluate Exo70 expression. RKO cells exhibited much higher protein and mRNA expression levels of Exo70 than the other three cell lines HCT116, HT29 and SW620 (Fig. 5a,b). Therefore, we overexpressed Exo70 in HCT116 cell line with low Exo70 expression, and applied shRNA to knockdown Exo70 expression in Exo70highly-expressed RKO cell line. Successful cDNA-mediated overexpression of Exo70 in HCT116 cells and shRNA-mediated knockdown of Exo70 (shExo70, using two different sequences namely S1 and S2) were confirmed by western blot analysis (Fig. 5c,d). To get insight into the potential roles of Exo70 as an oncogene that might influence the migration of the colon cancer cells, we evaluated the migration potential in HCT116 cells transfected with Exo70 cDNA and RKO cells transfected with shExo70. In the transwell migration assays, overexpression of Exo70 promoted the migration of HCT116 cells (Fig. 5e, *P* = 0.03). In contrast, knocking down Exo70 led to a reduction in the migratory property of RKO (Fig. 5f, shExo70-S1 *P* = 0.028, shExo70-S2 *P* = 0.003). Collectively, these data from *in vitro* experiments showed that Exo70 promoted the migration ability of colon cancer cells.

## Discussion

Metastatic recurrence is the leading cause of cancer death in patients with colon cancer. Conventional staging is insufficient to adequately predict metastasis or recurrence in colon cancer. The biological mechanisms driving poor clinical outcomes remain incompletely understood. Therefore, it is necessary to develop prognostic factors and novel therapeutic targets for the treatment and prevention of colon cancer. In the current study, real-time PCR, western blot and IHC analyses revealed increased levels of Exo70 in colon cancer tissues compared with adjacent non-malignant tissues. These results demonstrated that Exo70 could be a potential factor for colon cancer.

In mammals, the components of the Exocyst complex have a number of specific functions such as cell migration, host defense, nanotube formation, autophagy, ciliogenesis, apoptosis, DNA repair, pre-mRNA splicing and EMT. Because of this, a number of recent studies have implicated the mammalian Exocyst complex in the carcinogenesis of many different types of tumors^18^. Exo70, a key component of the Exocyst complex has been demonstrated to be important in human tumors correlates with cancer progression beyond exocytosis in recent literatures^19–23^. A key feature of cancer is the ability of the tumor cells to break through tissue barriers and invade into surrounding tissues^24^. The initial step of tumor cell invasion is the formation of cell protrusions in the direction of cell movement^25^. Exo70 plays a key role in migration, invadopodia formation and invasion^15, 16, 22^. Exo70 induces extensive membrane protrusions resembling filopodia. This effect is specific for Exo70 as none of the other Exocyst components are able to induce such actin structures. Blocking Exo70 function inhibits invadopodia formation^16^. Knockdown of Exo70 in MDA-MB-231 cells abolished the secretion of MMPs, whereas the overexpression of Exo70 promoted MMP secretion^22^. From HeLa cells and MDA-MB-231 cells, Exo70 directly interacts with the Arp2/3 complex, a core nucleating factor for the generation of branched actin networks for cell morphogenesis and migration^21^. And this interaction is important for the regulation of actin assembly at the leading edge of migrating cells^16^. Exo70 may be involved in tumor invasion by regulating MMP-9 secretion through synergistic reaction with CTTN in HCC^26^. EMT, a developmental program, is also critical for tumor cell dissemination and metastasis^27–29^. Selective expression of a single Exo70 isoform affects the expression of transcription factors such as Snail and ZEB2 and leads to cellular transitions between epithelial and mesenchymal phenotypes^17^. Exo70 knockdown can inhibits melanomas metastasis in nude mice^30^. Taken together, these studies suggest that Exo70 is implicated in several key stages of cancer dissemination.

However, to the best of our knowledge, this is the first study to evaluate the diagnostic and prognostic value of the expression of Exo70 in colon cancer patients. Here, we attempted to illustrate the relationship between Exo70 expression and clinicopathological features of colon cancer, especially the prognosis significance. In the present study, we found that Exo70 expression was significantly elevated in human colon cancer tissues. Initially, Exo70 expression was assessed at mRNA and protein level. Our real-time PCR results confirmed that higher Exo70 mRNA expression was detected in colon cancer tissues than in matched noncancerous tissues. Furthermore, Exo70 protein level, as analyzed by western blot and IHC showed that Exo70 expression was elevated in 92.3% (12/13) and 61.8% (55/89) of colon cancer tissues respectively. Taken together, these observations indicated an oncogenic role of Exo70 in colon tumorigenesis.

In the present study, IHC data showed that Exo70 was predominantly located in the cytoplasm both in normal colonic epithelial cells and colon cancer cells. However, whether Exo70 is functional in the malignant progression of human colon tissues needs further investigation. Our study revealed that the increased Exo70 expression in colon cancer was correlated with several worse clinical features, including tumor size, the depth of tumor invasion, distant metastasis, advanced TNM stage and worse OS (regardless of clinical stage), which further confirm a prooncogenic effect of Exo70. This indicated that Exo70 expression may be closely related to the invasion and metastasis of colon cancer.

Human cells have five Exo70 isoforms that differ in a region located in the middle of the proteins, isoform 1, 2, 3, 4, 5^17^. Interestingly, a recent report indicated that there were two different types of functional opposing Exo70 isoforms in breast cancer: Exo70-E (i.e., isoform 5, inhibits invasion/migration) and Exo70-M (i.e., isoform 2, increases invasion/migration)^17^. During EMT, Exo70-E goes down and Exo70-M goes up^17^. To explore whether such situation also exists in colon cancer, we induced EMT with TGF-β in several colon cancer cells including HCT-116, SW620 and RKO and observed the mRNA changes of Exo70 isoform 1, 2 (M form in breast cancers), 4 and 5 (E form in breast cancers). The results (Supplementary Fig. S2) showed that the mRNA levels of isoform 2 and 5 were not quite changed. However, the mRNA levels of Exo70 isoform 1 and 4 were significantly raised during EMT in these colon cancer cells. Nevertheless, none of these isoforms were down-regulated during EMT, suggesting an upregulation of total Exo70 in colon cancer cell, which is quite different from the situation in the breast cancer^17^. In fact, cancer originated from diverse tissues may have different Exo70 patterns, e.g., Exo70 in melanoma appeared as a single band (isoform)^30^ suggesting another pattern. The lack of Exo70 isoform switching in some cancer is probably because the M form of Exo70 (might be any isoforms) is easily to be upregulated, so there’s no need for the E form of Exo70 to be switched to M form. The roles of different Exo70 isoforms will be further studied in our future work. Therefore, we still propose that total Exo70 might be a promising negative prognostic factor and a potential therapeutic target for colon cancer, based on our observations and medical statistical analyses in this study.

Interestingly, the results of the current study demonstrated a significant correlation between Exo70 overexpression and shortened OS in our population. Univariate analysis showed that the increased Exo70 expression was significantly associated with the overall survival rate in colon cancer patients. Multivariate analysis indicated that Exo70 expression was an independent risk factor for poor prognosis of colon cancer patients. Thus, patients with a high level of Exo70 expression could be recommended to receive aggressive radiotherapy and chemotherapy to reduce tumor malignancy and metastasis. We also found that the overall survival is poor in colon cancer patients with increased Exo70 expression in stage II and stage II-III patients. Maybe Exo70 can be a risk factor for stage II disease when determining if adjuvant therapy should be administered. We also found that the overall survival is poor in colon cancer patients with increased Exo70 expression and lymphovascular invasion in stage II and stage II-III patients. Patients with high-risk stage II disease, are defined as with poor prognostic features, including T4 tumors (stage IIB/IIC), poorly differentiated histology (exclusive of those cancers that are MSI-H), lymphovascular invasion, PNI, bowel obstruction, lesions with localized perforation or close, indeterminate, orpositive margins, or inadequately sampled nodes (<12 lymph nodes). These patients can be considered for adjuvant chemotherapy with 5-FU/LV, capecitabine, FOLFOX, capecitabine/oxaliplatin (CapeOx), or bolus 5-FU/LV/oxaliplatin (FLOX)^31^. In our study, the results suggest that Exo70 may be a risk factor for stage II disease when determining if adjuvant therapy should be administered.

However, the main limitation of this study is the representative nature of our cohort and its retrospective nature. We found that most cases censored are in the moderately tumor differentiation group. Molecular classification of colon cancer, a heterogeneous disease, defines four different subtypes: CMS1 (MSI Immune), CMS2 (Canonical), CMS3 (Metabolic) and CMS4 (Mesenchymal)^32^. The heterogeneity of colon cancer cells introduces significant challenges in designing effective treatment strategies. It’s really a great pity that we did not have the opportunity to study Exo70 expression in different subtypes to interpret the colon cancer heterogeneity in our current cohort, because this molecular classification is not yet recommended in clinical practice (i.e., no information about the molecular classification of our current clinical samples was provided) and our study is retrospective.

It was proposed that secretion from stroma [exocytosis] is just as important, if not more for cancer invasion. So we have tried to study the relationship between our current cohort stromal staining and outcome. Our results showed that IHC staining with the anti-Exo70 antibody was specifically distributed in the cytoplasm/membrane of the tumor cells and their adjacent mucosal epithelial cells. Most of the positive stromal staining cells were inflammatory cells. However, the staining grades (IHC score) of the positive stromal staining cells were not considered to be comparable to that of the tumor cells and their adjacent mucosal epithelial cells. So it may be another limitation that we could not interpret the relationship exists between stromal staining grades and outcome in this study.

## Conclusion

In conclusion, our results demonstrate for the first time that increased Exo70 expression is associated with poor survival in colon cancer patients, suggesting that Exo70 expression may serve as an important prognostic factor and may represent a potential molecular target for the prevention and treatment of colon cancer. However, further study will be required to uncover and understand its mechanisms, processes, and interrelationships in order to determine how Exo70 is relevant to the etiology of colon cancer.

## Materials and Methods

### Patients, tissue specimens and follow-up

A total of 102 specimens were used for this study. The study was approved by the Medical Ethics Committee of Zhongshan Hospital Affiliated to Xiamen University in accordance with the Helsinki Declaration and conducted with the informed consent of all patients. All tissue samples were obtained from the tissue bank of Zhongshan Hospital Affiliated to Xiamen University. Patients who were diagnosed with cancers of any other histotype and those with a family history of colon cancer were excluded from the study.

Formalin-fixed and paraffin-embedded primary colon cancer and matched adjacent nontumor colon tissues from 89 patients were collected for tissue microarray (TMA) construction. TMAs were constructed by ALPHELYS MiniCore series 3; 1-mm cores from donor blocks were transferred into a recipient block. The matched normal colon tissues were obtained from a segment of the resected specimens that was >5 cm away from the tumor. None of the patients received preoperative radiation or chemotherapy. In addition, another13 paired tissues obtained immediately after surgery were snap-frozen in liquid nitrogen and kept at −80 °C for further analysis. So there are totally 102 samples, in which 89 matched samples are for TMA analysis and 13 samples are for analysis for mRNA and protein expression. The demographic and clinicopathological information for all the colon cancer cases, including age, sex, tumor size, tumor location, tumor differentiation, depth of invasion, lymphovascular invasion, lymph node metastasis, distant metastasis and TNM stage, were simultaneously collected from each patient’s medical records. Pathological examination of a minimum of 12 lymph nodes was asked in all cases. Cancers were staged according to the AJCC Colon Cancer Staging, 7^th^ edition (2010). Complete follow-up data were available until March 2016 for all colon cancer patients. Overall survival (OS) was calculated from the date of resection to the date of death or last follow-up.

### Real-time PCR

Total RNA was extracted from fresh frozen colon cancer tissues and the matched noncancerous tissues using Trizol reagent (Invitrogen, Carlsbad, CA, USA) and then reverse-transcribed into cDNA using Primescript™ RT reagent kit (A5000; Promega, Madison, Wisconsin, USA) according to the manufacturer’s instructions. Real-time PCR was then carried out using the SYBR Green I fluorescent dye (SYBR® Premix Ex Taq™ II, TaKaRa, Dalian, China) and the StepOnePlus™ real-time PCR system (Applied Biosystems, Australia). The thermal cycling consisted of an initial pre-degeneration at 95 °C for 30 s, followed by 40 cycles of de-naturation at 95 °C for 5 s and annealing/extension at 60 °C for 30 s. The glyceraldehyde 3-phosphate dehydrogenase (GAPDH) mRNA level was used to standardize the measurements ofExo70. Primers used were listed as follows:

Exo70, 5′-GGAGTATTTCCAGGACAACAGC-3′ (forward),

5′-AAGATGAGCACGGGCGAGA-3′ (reverse);

Exo70-isoform 1, 5′- ATTCCTCTGGAAGGGAGAGAT-3′ (forward),

5′-GAGCTGGTCTCTTGGGAGG-3′ (reverse);

Exo70-isoform 2, 5′- CAAGCGGCCAGGGAGAGATGAC-3′ (forward),

5′- GGCATTGCTGGTGAGCTCGTGTA-3′ (reverse);

Exo70-isoform 4, 5′- TAAACGCTGCTTGTGTTTGT-3′ (forward),

5′- ATGTAGGCATCGGTCTCC-3′ (reverse);

Exo70-isoform 5, 5′- TGGCCGCAACCAAGATTTCATG-3′ (forward),

5′- TCGGACAGGTGCTTAACTCGGAAAT-3′ (reverse);

GAPDH, 5′-TGCACCACCAACTGCTTAGC-3′ (forward),

5′-GGCATGGACTGTGGTCATGAG-3′ (reverse);

Relative mRNA levels were quantified by the comparative 2^−ΔΔCt^ method. The experiments were independently repeated three times.

### Western blot

Total protein was extracted by using a lysis buffer and protease inhibitor (Beyotime Biotechnology, China). Equivalent protein amounts were denatured in an SDS sample buffer, and then were separated by SDS-PAGE and transferred onto polyvinylidene difluoride membrane. After being blocked with 5% non-fat dry milk in PBS containing 0.05% Tween-20, the blotted membranes were incubated with anti-human Exo70 antibody (1:4000, Abcam, Cambridge, MA, USA) and then secondary antibody (1:5000, Boster, China). β-actin protein levels also were determined by using the specific antibody (1:3000, Abcam, Cambridge, MA, USA) as a loading control. Immunoreactive bands were detected using Enhanced Chemiluminescence (ECL) system (Bio-Rad, Hercules, CA, USA).

### Immunohistochemical analysis

Immunohistochemical analysis was performed using formalin-fixed and parrffin-embedded (FFPE) colon cancer cell pellet slide and colon cancer tissue sections. FFPE cell pellet slide: fresh, 5-μm-thick sections were placed on coated glass slides, deparaffinized, and rehydrated. Expression of Exo70 was determined by Immunohistochemistry (IHC) staining of the FFPE cell pellet slides and tissue sections using mouse anti-Exo70 monoclonal antibody (ab57402, Abcam, Cambridge, MA, USA). IHC was performed using a standard avidin-biotin-peroxidase complex method. Four-micrometer thick sections of TMAs mentioned previously were deparaffinized in xylene, dehydrated in gradient concentrations of ethanol, and then subjected to high-pressure antigen retrieval in a pressure cooker for 3 min in preheated 10 mmol/L sodium citrate buffer (pH 6.0). Endogenous peroxidase activity was blocked by incubation in 3% hydrogen peroxide for 10 min, and nonspecific staining was eliminated by incubating the sections with normal goat serum for 15 min at room temperature. The sections were incubated with the primary antibody (ab57402, 3 μg/ml) at 4 °C overnight. After being washed with phosphate-buffered saline, sections were incubated with diluted biotinylated goat anti-rabbit secondary antibody for 10 min and then incubated with the avidin-biotin-peroxidase complex for another 10 min with repeated washing steps. Staining was visualized using 3,3′-diaminobenzidine solution (Maxim, Fuzhou, China). Sections were then counterstained with hematoxylin after dehydration and clearing with xylene. Coverslips were added to the slides and examined. Negative controls were obtained by omission of the primary antibody. A final agreement was obtained for each score using a multi-head microscope (Olympus BX51, 10-headed microscope).

### Evaluation of immunohistochemical staining

Immunohistochemical staining was blindly scored by two pathologists. The results of most samples scored by two pathologists in this study were accordance with quite little difference, and the average score were taken as the final results. Few samples got discordance results were rescored by two pathologists, and the results agreed by both of them were taken as final results used in the statistical analysis. The Exo70 protein was assessed in both the cytoplasm and nucleus. The overall amount of staining was determined by the staining intensity and the proportion/extent of stained tumor cells according to published scoring methods^33, 34^. The mean percentage of positive tumor cells was determined in at least five areas at 400x magnification and assigned to one of the following categories: 0, ≤5%; 1, 5–25%; 2, 25–50%; 3, 50–75%; 4, ≥75%. The average estimated intensity of staining in positive cells was scored as 1 (weak), 2 (moderate) or 3 (intense). The total score was calculated by multiplying the scores for intensity and the extent of staining. Based on evaluation score, the expression level of Exo70 was classified into: 0 point, negative (−); 1~4 points, weakly positive (+); 5~8 points, moderate positive (++); 9~12 points, strongly positive (+++). The “+”, “++”, “+++” were regarded as positive signals with observable increase in staining intensity.

### Cell culture and transfections

The human colon cancer cell lines HCT116, HT29, RKO and SW620 were purchased from ATCC (Manassas, USA) and cultured in Dulbecco’s Modified Eagles Medium (DMEM, Lonza, Basel, Switzerland). All the medium was supplemented with 10% fetal bovine serum (HyClone, Logan, UT, USA), 100 U of penicillin, and 100 μg/ml of streptomycin (Life Technologies, Carlsbad, CA, USA), and propagated at 37 °C in a 5% CO_2_ humidified atmosphere. The cells were used within 15–20 passages after the initiation of cultures. Cell transfections were carried out with the Lipofectamine 2000 transfection reagent (Invitrogen) according to the manufacturer’s instructions.

### Plasmids

To reveal the effects of Exo70 on the ability of migration, an Exo70 expression vector and two shRNA plasmids targeting Exo70 (shExo70) were constructed. Targeting sequence for shExo70-S1: AAGATTTCATGAACGTCTA; targeting sequence for shRNA-S2: TGCAGGAGAATGTTGAGAA; targeting sequence for negative control: TTCTCCGAACGTGTCACGT. Oligonucleotides (Invitrogen, Guangzhou, China) were annealed and inserted into pGV248 (for shExo70). Rattus Exo70 (kindly provided by Wei Guo, University of Pennsylvania, Philadelphia, USA) was separately subcloned into pCMV10/3× FLAG (Sigma-Aldrich, St. Louis, MO, USA). They were transfected separately into RKO and HCT116 cells by Lipofectamine 2000 (Invitrogen, Carlsbad, CA, USA).

### Migration Assay

Cell migration assays were performed using 8.0 mm pore size transwell chamber (Corning USA). 2 × 10^4^ cells suspended in 200 μl serum-free medium were added into the upper transwell chamber, and 500 μl medium supplemented with 10% serum was placed in the bottom chamber at 37 °C in a humidified atmosphere of 5% CO_2_. After 24 h incubation, cells on the upper surface were removed with a cotton swab. Cells invading to the lower surface of membrane were fixed with methanol before staining with 0.4% crystal violet for 30 min. The stained cells were counted in 5 randomly selected fields under an inverted microscope.

### Statistical analysis

Associations between Exo70 expression and clinicopathologic variables were analyzed by Pearson Chi-Square test. The median OS, as well as their 95% confidence intervals (95% CIs), were estimated by the Kaplan-Meier method. The difference in survival was analyzed using the log-rank test. Univariate and multivariate Cox regression analyses were performed to identify the factors that had a significant influence on survival. The differences in Exo70 mRNA expression between carcinoma and normal samples were evaluated by the independent-sample *t* test. Results are expressed as the mean ± SD and the data are representative of at least three separate experiments. The diagnostic value of Exo70 in discriminating Colon cancer was confirmed by receiver-operator characteristic (ROC) curves analysis using the 0,1-criterion. The ROC curves were generated by plotting the sensitivity and specificity for each outcome at various Exo70 scores. The score closest to the point (0.0, 1.0) on the curve, with maximum sensitivity and specificity, was selected as the cut-off value. A *P* value less than 0.05 (two-sided) was considered to be statistically significant. All statistical analyses were conducted using SPSS17.0 statistical software (SPSS Inc, Chicago, IL, USA).

## Electronic supplementary material

Supplementary information
