# Supplementary material for: Exo70 is an independent prognostic factor in colon cancer
Source: Sci Rep. 2017 Jul 11;7:5039. doi: 10.1038/s41598-017-05308-x (PMC5505949; doi:10.1038/s41598-017-05308-x)

Supplementary information

Exo70 is an independent prognostic factor in colon cancer

Li Xiao[1,2,ξ], Kaifeng Zheng[1,ξ], Xia Lv[2], Jihuan Hou[1], Liang Xu[1], Yujie Zhao[1], Fei Song[1], Yaqiong Fan[1], Hanwei Cao[1], Wenqing Zhang[1], Xiaoting Hong[1], Yan-yan Zhan[1,*], and Tianhui Hu[1,*]

[1]Cancer Research Center, Xiamen University Medical College, Xiamen 361102, Fujian Province, PR China

[2]Department of Oncology, Zhongshan Hospital Affiliated to Xiamen University, Xiamen 361004, Fujian Province, PR China

[ξ]These authors have contributed equally to this work

***Correspondence to:** Tianhui Hu, e-mail: thu@xmu.edu.cn or Yan-yan Zhan, email: yyzhan@xmu.edu.cn

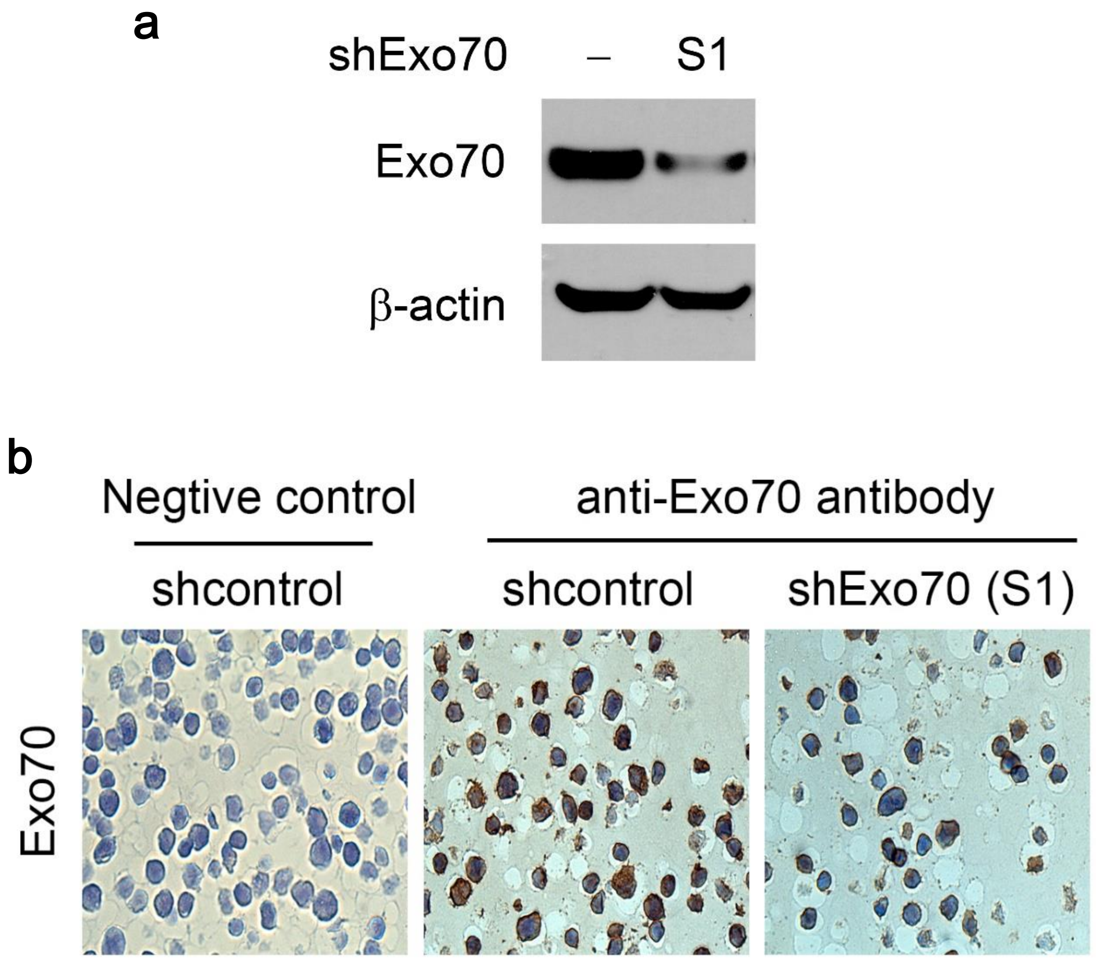

Supplementary Figure S1. Verification of the specificity of anti-Exo70 antibody by immunohistochemistry on formalin-fixed and parrffin-embedded (FFPE) EXO70 knocking-down colon cancer cell pellet slide. (a-b) RKO cells were transfected with shRNA (S1 mentioned in Fig. 5) targeting Exo70, and then subjected to western blot (**a**) and immunohistochemical staining (**b**) using anti-Exo70 antibody (Abcam, ab57402)**.**

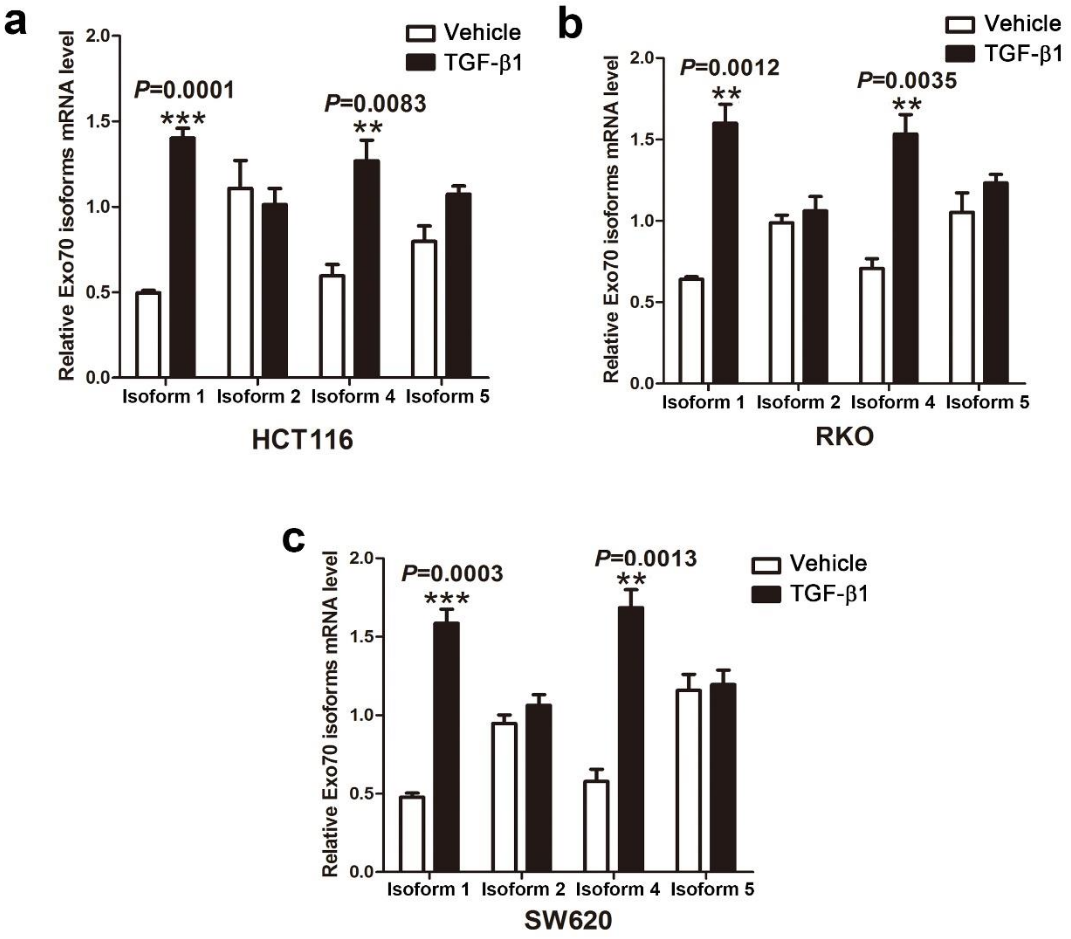

Supplementary Figure S2. Expression levels of Exo70 isoforms during EMT in colon cancer cells. (a-c) HCT116, RKO and SW620 cells were treated with human TGF-β1 (20 ng/mL) for 72h, and then subjected to qRT-PCR. $^{**}P<0.01$, $^{***}P<0.001$.

Supplementary Figure S3. Original western blots

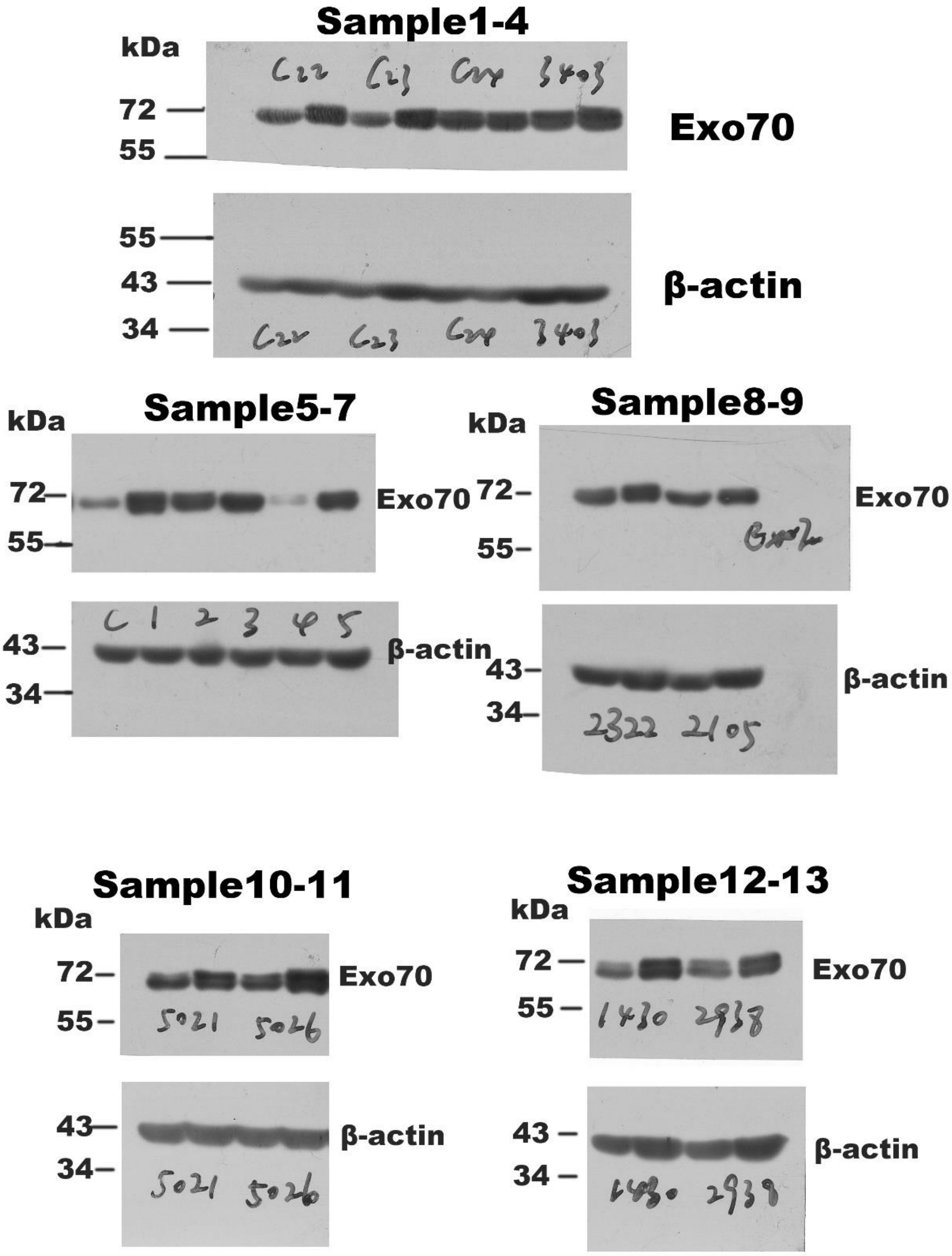

Supplementary Figure S4. Original western blots

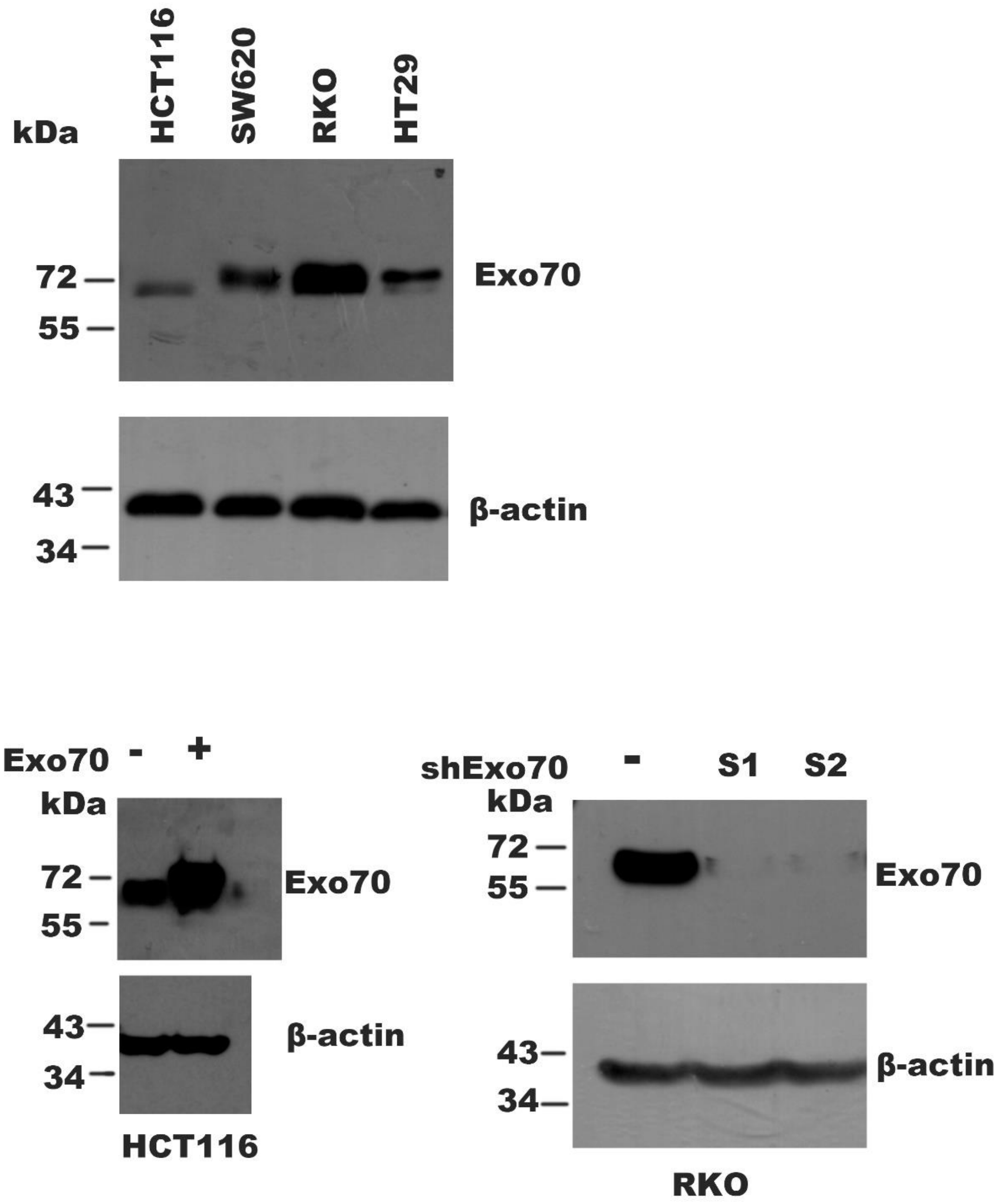

Supplement: Supplementary file 1 — Supplementary information [file 41598_2017_5308_MOESM1_ESM.pdf]
